# Efficacy, Safety, and Cost-Effectiveness of Cell Salvage in Cardiac Reoperations

**DOI:** 10.5152/eurasianjmed.2026.251304

**Published:** 2026-06-09

**Authors:** Osman Fehmi Beyazal, Nihan Kayalar, Mehmed Yanartaş

**Affiliations:** Department of Cardiovascular Surgery, Başakşehir Çam and Sakura City Hospital, İstanbul, Türkiye

**Keywords:** Autologous, cell salvage, cost-effectiveness, reoperation, transfusion

## Abstract

**Background:**

The aim of this study was to investigate the efficacy and safety of cell salvage (CS) use in cardiac reoperations and to conduct a cost analysis considering the Turkish economy.

**Methods:**

Between 2024 and 2025, a total of 45 patients who had undergone cardiac reoperations were included in the study. The patients were divided into 2 groups: group A (n = 31, CS used) and group B (n = 14, CS not used). Intraoperative and postoperative blood usage, as well as all postoperative outcomes, were compared between the groups. Cost analysis was conducted by comparing the costs of blood products and CS.

**Results:**

There were no differences between groups A and B in terms of baseline demographic characteristics, comorbidities, EuroSCORE II, NYHA stages, and operative data. The need for intraoperative and postoperative red blood cells (RBCs), fresh frozen plasma (FFP), and apheresis platelet suspension (APS) was lower in group A, but there was no statistical difference. The amount of bleeding and all postoperative outcomes were similar between the groups. Although intraoperative and postoperative RBCs, FFP, and APS costs were lower in group A than in group B, there was no statistical difference. However, when CS cost was added, the total blood product cost and CS cost were significantly higher in group A than in group B (mean $448 - $240, *P* < .001, respectively).

**Conclusion:**

It was found that the routine use of CS in cardiac reoperations is not cost-effective in Türkiye, but it is safe.

**Main Points**

- To the best of present knowledge, this is the first study to evaluate the cost-effectiveness of cell salvage on cardiac reoperations.
- Intraoperative and postoperative blood product use and the amount of bleeding were statistically similar in patients with and without cell salvage.
- The routine use of cell salvage in cardiac reoperations is not cost-effective in Türkiye, but it is safe.
- Due to its potential to reduce blood transfusion requirements, intraoperative decisions should be made in selected patients.

## Introduction

Cardiac surgery is a discipline that involves significant blood loss and often requires blood transfusions. Many factors contribute to the risk of bleeding in cardiac procedures, including the nature of the surgery itself, the effects of cardiopulmonary bypass (CPB), the use of heparin during CPB, and the administration of preoperative and postoperative antiplatelet/anticoagulant medications. Despite advancements in equipment and surgical techniques aimed at reducing blood loss, cardiac surgery remains one of the medical fields with the highest blood product consumption.

The use of cell salvage (CS) has become increasingly widespread in recent years to reduce blood loss, prevent unnecessary transfusions, and decrease the risk of associated complications. With CS, lost blood is filtered, washed, and then recycled back to the patient, allowing for significant blood salvage. However, the literature contains limited data and conflicting results regarding the effectiveness, safety, and, especially, cost analysis of these systems.

It has been reported that the CS may have adverse effects on coagulation due to the centrifugation and washing procedure.^1^ Wang et al reported that when the amount of bleeding was between 600-1000 mL, the need for blood was significantly reduced with CS. It had no direct negative effect on coagulation functions and was cost-effective.^2^ Xie et al reported that CS is effective and cost-effective in developed countries for cardiac surgery with a high risk of bleeding, but it is not cost-effective in China.^3^ In a study conducted by Klein et al in the United Kingdom (UK), it was reported that while CS reduces the need for blood products in cardiac surgery, it also leads to increased costs for the institution.^4^

Most studies on this topic have focused on cardiac surgery patients. Due to varying results regarding cost-effectiveness, many centers do not routinely use it in standard cardiac surgery procedures. However, its use has become widespread in patients with a high risk of bleeding, such as those undergoing reoperations, as it can reduce blood product requirements. Despite this, there is insufficient data on cost-effectiveness for cardiac reoperations. Additionally, many studies have been conducted outside of Türkiye, and local economic factors can impact the results of cost analysis. The aim of this study is to investigate the efficacy and safety of CS use in cardiac reoperations and to conduct a cost analysis considering the Turkish economy.

## Material and Methods

This study was designed as a retrospective, single-center, observational study involving a total of 45 patients. The study included all patients aged 18 years and older who had undergone cardiac reoperation at the Cardiovascular Surgery Clinic of İstanbul Başakşehir Çam and Sakura City Hospital between January 2024 and August 2025. Patients with aortic dissections, those who had used intraaortic balloon pump or extracorporeal membrane oxygenation, those who had undergone surgery while on dual antiplatelet therapy, and those who had delayed sternal chest closure were excluded from the study. The patients were divided into 2 groups: group A (n = 31, CS used) and group B (n = 14, CS not used).

Medical records of all patients were reviewed including basic demographic characteristics, medical history, preoperative and postoperative first week transthoracic echocardiographic (TTE) findings, preoperative and postoperative 1st day laboratory parameters, surgical procedure details, cross clamp (XCL) times, cardiopulmonary bypass (CPB) times, activated clotting time (ACT) values, vasoactive inotrope scores (VIS), the European System for Cardiac Operative Risk Evaluation (EuroSCORE II) score, New York Heart Association (NYHA) stages, bleeding amount, blood product use, blood product costs, CS cost, postoperative complications (postoperative exploration, cerebrovascular accident (CVA), postoperative atrial fibrillation (POAF), continuous renal replacement therapy (CRRT) need, thoracentesis, implantable cardioverter defibrillator (ICD) need, gastrointestinal bleeding, tracheostomy, mortality, intubation time, intensive care unit (ICU) stay, and hospital stay. Comparisons were then made between groups A and B regarding all these parameters. Intraoperative and postoperative blood product usage amounts were determined, and costs were calculated separately and in total for blood product usage based on the exchange rate prevailing at the study date. The current cost of the blood transfusion was added to these prices, and the total cost difference between groups A and B was compared. Costs were converted from Turkish Lira to United States dollars, considering the most recent exchange rate. In the clinic, the Sorin Xtra® Autotransfusion System is used as the blood transfusion device. The current price for the Autotransfusion System was $240.9. The costs per unit of red blood cells (RBCs), fresh frozen plasma (FFP), and apheresis platelet suspension (APS) were $34.8, $20.6, and $113.2, respectively.

In the clinic, CS is not routinely used in cardiac surgery. In cardiac reoperations, where the bleeding risk is higher than with standard procedures, the decision to use CS routinely is based on the type of surgery, the bleeding risk, and the surgeon’s preference. The threshold value for erythrocyte transfusion is set at <8 g/dL.

This study was approved by the İstanbul Başakşehir Çam and Sakura City Hospital Ethics Committee (Decision no: 2025-320, September 9, 2025). The study was conducted in accordance with the Declaration of Helsinki. Patients’ informed consent was obtained for this study. Artificial intelligence-assisted technologies were not used in the production of the submitted work.

### Statistics

Statistical analyses were performed using IBM SPSS Statistics for Windows, Version 20.0 (IBM SPSS Corp.; Armonk, NY, USA). Continuous variables in the study are presented as minimum, maximum, median, and interquartile range. Categorical variables are expressed as numbers and percentages. The normality of distribution was assessed by the Kolmogorov–Smirnov test. For numerical variables, differences between patients and controls were tested using t test for parametric data or the Mann–Whitney *U-*test for non-parametric data. Categorical variables were analyzed using the Pearson χ^2^ test and Fisher’s exact test. The level of statistical significance was set at *P* < .05.

## Results

The patient’s demographics, comorbidities, and TTE findings are presented in Table 1. The mean age was 53.3 ± 16.1 years, with 23 (51.1%) female patients. The mean follow-up period was 377.8 ± 209 days (median: 407, 1-635 days). There was no difference between groups A and B in terms of baseline demographic characteristics, comorbidities, and NYHA stages. Additionally, there was no difference in EuroSCORE II scores between the groups (mean, 20.4%-18.6%, respectively, *P* = .75). Postoperative VIS was also similar between the groups. Preoperative ejection fraction (EF) was higher in group A than in group B (median, 60%-55%, respectively, *P* = .001). There was no difference in preoperative tricuspid annular plane systolic excursion (TAPSE) between the groups. Postoperative EF and TAPSE values were similar between the groups.

A comparison of operative data, drainage volume, blood product use, and intraoperative medications is presented in Table 2. The rates of emergency surgery, coronary artery bypass grafting (CABG), valvular surgery, aortic surgery, infective endocarditis, the Commando procedure, ablation, and congenital anomaly surgery were similar between the groups. There was no difference between the groups in terms of XCL and CPB times. Intraoperative RBCs, FFP, and APS use were lower in group A than in group B, although not statistically significantly different (mean 1.8-1.9, *P* = .91, 0.9-1, *P* = 0.77, 0.6-0.8, *P* = 0.63, respectively). Similarly, postoperative RBCs, FFP, and APS use were less in group A than in group B, although no statistical difference was found (mean 0.9-1, *P* = 0.30, 0.2-0.5, *P* = .68, 0.06-0.07, *P* = .93, respectively). Postoperative bleeding was also less in group A than in group B, although no statistical difference was found (mean 768-785 ml, *P* = 0.68, respectively). Intraoperative tranexamic acid use was also similar between the groups. No difference was found between the groups in terms of ACT values after protamine.

A comparison of laboratory parameters between the groups is presented in Table 3. No differences were found between groups A and B regarding all detailed preoperative laboratory parameters. Postoperative day 1 white blood cell (WBC), hematocrit, platelet, urea, creatinine, sodium, potassium, alanine aminotransferase (ALT), C-reactive protein, and troponin T values were similar between the groups. The only difference was that aspartate aminotransferase (AST) was higher in group A than in group B.

A comparison of postoperative outcomes between the groups is presented in Table 4. There were no differences between the groups in terms of postoperative exploration, POAF, need for CRRT, thoracentesis, need for ICD, gastrointestinal bleeding, or tracheostomy. No CVA was observed in either group. There was no statistically significant difference between groups A and B in terms of mortality rates (6 (19.4%)-1 (7.1%), respectively, *P* = .28). Additionally, no significant differences were found between the groups in terms of postoperative intubation time, ICU stay, or hospital stay.

**Figure 1.**
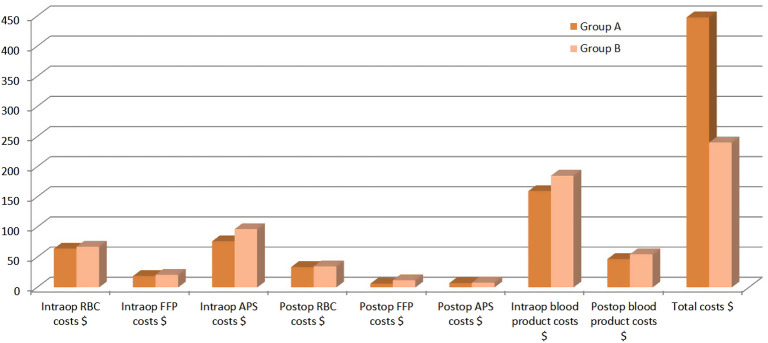
A comparison of the overall cost analysis between groups A and B.

A comparison of costs between groups is presented in Table 5. Intraoperative costs for RBCs, FFP, and APS were lower in group A than in group B, although no statistically significant difference was found (mean $64.3-$67.5, *P* = .91, $18.9-$21, *P* = .77, $76.5-$96.8, *P* = .63, respectively). Similarly, postoperative costs for RBCs, FFP, and APS were also lower in group A than in group B, although no statistically significant difference was found (mean $33.8-$35, *P* = .30, $6.1-$12, *P* = .68, $7.2-$8, *P* = .93, respectively). Total intraoperative and postoperative blood product costs were also lower in group A than in group B, although not statistically significantly different (mean $159.8-$185.3, *P* = .51, $47.2-$55, *P* = .27, respectively). The total blood product cost and CS cost were significantly higher in group A than in group B (mean $448-$240, *P* < .001, respectively). A comparison of the overall cost analysis between the groups is shown in Figure 1. A cost-effectiveness comparison of studies in different countries is also presented in Table 6.

## Discussion

There are few studies that analyze the effectiveness, safety, and cost of CS use in cardiac surgery, considering the Turkish economy. To the best of present knowledge, there is no research on this topic in cardiac reoperations. In this study, it was observed that CS use in cardiac reoperations in Türkiye is not cost-effective, but it is safe and may be effective in reducing the need for blood transfusions.

Transfusion reactions are clinical conditions that can occur either acutely or after days or weeks. They can range from mild to life-threatening and may be hemolytic or non-hemolytic, anaphylactic, simple allergic, septic, or transfusion-related acute lung injury.^5^Cardiac surgery accounts for a significant portion of blood transfusions worldwide. In the UK, approximately 10% of all blood provided by the National Blood Service is reported to originate from cardiac procedures, with at least 3-quarters of patients receiving at least 1 unit of blood transfusion.^6^ Suleman et al reported that approximately 47% of patients undergoing elective CABG required intraoperative transfusion.^7^ Another study reported that up to 92% of elective cardiac surgery patients received blood transfusions.^8^ In this study, 36 (80%) patients received at least 1 unit of RBCs either intraoperatively or postoperatively. Due to the scarcity and cost of blood products, as well as the risks associated with transfusion, there has been a growing interest in blood conservation during cardiac surgery. As a result, numerous articles have been published on patient blood management in cardiac surgery.^9^^,^^10^ However, due to the nature of cardiac surgery, patients may still require blood product transfusions.

In cardiac surgery, bleeding that occurs after the initiation of CPB is directed to the reservoir, thus avoiding significant postoperative losses. However, bleeding before and after CPB cannot be transferred to the reservoir, making bleeding during these stages a primary cause of blood loss. In recent years, many centers have been utilizing CS to reduce the need for blood transfusions. The RBCs collected have a hemoglobin and hematocrit concentration above 50%.^11^ It is believed that by doing so, the need for blood transfusions can be reduced along with the associated risk of reactions.

On the other hand, the main disadvantages of the CS include the washing away of platelets and plasma content, destruction of cell components during blood recovery and centrifugation, absence of clotting factors, high concentration of heparin, and the potential to cause dilutional coagulopathy, fat and air embolism.^2^^,^^3^^,^^12^^,^^13^ It further activates the coagulation system, leading to a strong generation of thrombin. This directly depletes coagulation factors, activates platelets, and promotes fibrinolysis.^1^ Xie et al reported that the coagulation function of blood may be impaired by CS, potentially increasing the incidence of postoperative excessive bleeding.^3^ Murphy et al reported that there was no coagulopathic or thrombotic effect with CS and no increase in bleeding or thrombotic events.^14^ Goel et al also reported that the use of CS was not associated with a clinically significant bleeding diathesis.^15^ In the study, postoperative bleeding amounts, postoperative exploration rates, gastrointestinal bleeding rates, postoperative hematocrit and platelets, and postprotamine ACT values were similar between the groups. These results suggest that CS use does not significantly increase the risk of bleeding.

Another important point about CS is its effectiveness. Niranjan et al reported that CS significantly reduced transfusions in cardiac surgery.^13^ Weltert et al also reported that the use of CS is a safe and effective strategy to reduce allogeneic RBC transfusions.^16^ On the other hand, Reyes et al reported that the need for blood transfusion was not reduced by CS in low-risk patients.^17^ Klein et al also reported that the use of CS did not reduce the number of patients requiring blood transfusions during initial non-emergency cardiac surgery.^4^ In the study, both intraoperative and postoperative RBCs, FFP, and APS utilization were lower in the CS group than in the control group, but there was no statistical difference. The main reason for the decreasing trend in the need for blood transfusions may be the fact that blood lost due to bleeding before and after CPB can be recovered and returned to the patient using the CS. These results suggest that CS offers a partial benefit in blood product utilization, although it is not statistically significant.

One of the most debated issues regarding CS use is its cost. Few studies have been conducted on cost analysis, with most focusing on cardiac surgery patients and excluding reoperations.^2^^,^^4^^,^^14^ Cardiac reoperations, which are among the highest risk groups for bleeding in cardiac surgery, may benefit from the potential positive effects of CS on bleeding. However, when the cost of CS is added to this, a detailed cost analysis is necessary. One of the most important points regarding this cost analysis is the variation in cost factors across countries. Xie et al reported that the use of CS is cost-effective in developed countries but not in China.^3^ Klein et al also reported that the use of CS increased the cost per patient by at least $103.^4^ Attaran et al also reported that the routine use of CS in all cardiac surgeries did not provide any benefit and resulted in additional costs.^12^ Wang et al reported that the use of CS in off-pump CABG is cost-effective.^2^ Murphy et al also reported that the use of CS does not significantly increase costs and should be routinely used in off-pump CABG.^14^ Weltert et al reported that the use of CS resulted in a transfusion-related cost savings of $85 per patient in low-bleeding-risk patients.^16^ In this study, as shown in Figure 1, a decrease in the cost of all types of intraoperative and postoperative blood products were observed in the CS group. However, this difference was not statistically significant. When considering the cost of CS, it can be concluded that the CS group significantly increased the overall cost.

However, one of the most important factors affecting this situation is the economic differences between countries. RBCs and CS costs vary significantly across countries (Table 6). As in the country, the cost of RBCs in China is significantly lower than in countries like Italy and the UK. Cell salvage costs are higher in Italy, China, and Türkiye compared to the UK. While analyzing costs by calculating the cost of blood products and CS used gives an idea of cost-effectiveness, the difference between national economies cannot be ignored. In the study, the total mean cost was $448 in the CS group and $240 in the control group. The cost of 1 CS alone is equivalent to the cost of 6.9 units of RBCs. However, in a study by Klein et al in Britain, the cost of 1 CS is equivalent to the cost of 0.6 units of RBCs.^4^ In a study by Xie et al in China, the cost of 1 CS is equivalent to the cost of 10.6 units of RBC.^3^ In a meta-analysis by Pabon-Carrasco et al, the cost of CS for any cardiac surgery is equivalent to the cost of 2 units of RBCs.^11^ As seen, the cost of blood products and CS is closely related to the cost-effectiveness of CS. While cost analysis is crucial for healthcare policies, it should be noted that costs can vary across countries. Furthermore, factors such as the type of surgery and bleeding risk can influence costs. In patients with low bleeding risk, if the CS is installed but fails to collect sufficient volume, it can lead to a significant cost increase without any benefit. In the high-risk patient group in the study, a partial reduction in blood transfusion was observed in the CS group, although not statistically significant. Given the importance of patient blood management, it is believed that each reduced unit of transfusion will benefit the patient. Therefore, although not routine cardiac surgery, routine CS may be beneficial in procedures with high bleeding risk, such as reoperations. While not cost-effective in Türkiye, they may still benefit the patient.

Another important point to consider is the variability of transfusion thresholds across studies. In the study by Murphy et al the transfusion trigger was a hemoglobin concentration of <8 g/dL.^14^ In the study by Dalrymple-Hay et al the transfusion trigger was a hemoglobin concentration of <10 g/dL.^6^ Klein et al performed transfusions at intraoperative levels of <7 g/dL and postoperative levels of <8 g/dL.^4^ In the clinic, the threshold for RBC transfusion was set at <8 g/dL. These differing threshold values will result in varying numbers of blood transfusions and associated costs.

In addition, comparing postoperative outcomes related to CS use in cost analysis would be more beneficial. It would be valuable to investigate the long-term consequences of decreased blood transfusions and the resulting reduced transfusion reactions. Furthermore, examining the effects of CS use on postoperative exploration, intubation time, ICU stay, and hospital stay, as well as any potential cost differences, is also important. However, in the study, although preoperative EF was lower in group B, no significant difference between the groups were found in terms of all postoperative outcomes. Xie et al also reported no increase in postoperative adverse events. Wang et al similarly reported no difference in the duration of mechanical ventilation, ICU stay, or hospital stay.^3^ Djaiani et al reported a significant reduction in postoperative cognitive dysfunction after cardiac surgery with CS.^18^ In the study, no patients experienced postoperative CVA. Weltert et al reported lower rates of atrial fibrillation in the CS group.^16^ However, in the study, no difference between the groups were found in terms of POAF. Future studies with a larger number of patients could provide a better evaluation of postoperative outcomes with the use of CS.

Finally, the study group is considered a high-risk group for bleeding, as they have undergone complex surgeries, such as the Commando procedure and stuck valve surgery. In contrast, many studies on CS have excluded patients who have had reoperations.^2^^,^^4^^,^^8^^,^^12^^,^^13^^,^^17^ In the study by Reyes et al, patients with a Logistic EuroSCORE >10% were excluded.^17^ However, in this study group, approximately 50% of patients underwent emergency surgery. The mean EuroSCORE II values for groups A and B were 20.4%-18.6%. Postoperative exploration was observed in only 2 (4.4%) of the entire patient group. The observed mortality rates were also below the EuroSCORE II values in both groups. Based on these results, it can be concluded that reoperations in this high-risk patient group were performed with acceptable expected outcomes.

### Limitations

The most important limitation is that this was a single-center, retrospective study with a small, heterogeneous patient group. However, due to the infrequency of cardiac reoperations and the lack of sufficient studies on this topic, this study provides valuable results. Secondly, while it contains important findings for demonstrating inter-country differences for cost analysis, differences in study dates may affect costs. Thirdly, analysis was not possible due to the lack of records regarding the transfusion volumes administered with CS. Using CS may have resulted in a very small amount of blood being transfused, which may have been wasted. In cases where a large volume of transfusion was administered, postoperative coagulation may have been affected. Fourthly, intraoperative thromboelastography was not used when deciding on blood transfusion because it is not routinely performed in the clinic. Finally, early postoperative outcomes were compared. Studies specifically investigating the effects of blood transfusion in the medium-long term could shed light on this issue.

## Conclusion

In this retrospective study, it was found that the routine use of CS in cardiac reoperations is not cost-effective in Türkiye, but it is safe. However, due to its potential to reduce blood transfusion requirements, intraoperative decisions should be made in selected patients. Further studies with larger patient numbers are needed.

## Figures and Tables

**Table 1.** Comparison of Patient Demographics, Comorbidities, and Echocardiographic Findings Between Group A and Group B

	Group A (n = 31) Cell Salvage (+)			Group B (n = 14) Cell Salvage (−)			*P*
	Min-max or n (%)	Median (Mean)	IQR	Min-max or n (%)	Median (Mean)	IQR	
Demographic data							
Gender female	17 (54.8)			6 (42.9)			.45
Age (years)	18-84	54	19	21-77	57	12	.48
Height (cm)	150-183	168	13	156-190	170	15	.29
Weight (kg)	50-150	75	15	58-115	75	15	.90
Body surface area (kg/m^2^)	1.53-2.60	1.86	0.14	1.56-2.32	1.9	0.16	.67
Body mass index (m^2^)	17.3-46.2	27.1	7.02	21.9-35.4	25.3	5.4	.39
Comorbid diseases							
Diabetes mellitus	10 (32.3)			4 (28.6)			.54
Hypertension	16 (51.6)			5 (35.7)			.32
Chronic obstructive pulmonary disease	2 (6.5)			1 (7.1)			.68
Cerebrovascular accident	5 (16.1)			4 (28.6)			.28
Preoperative atrial fibrillation	8 (25.8)			4 (28.6)			.55
Chronic renal failure	1 (3.2)			0			.68
Thyroid disorder	4 (12.9)			1 (8.1)			.21
Rheumatic disease	1 (3.2)			0			.68
Preop NYHA stage	3-4	3 (3.2)	1	3-4	3 (3.3)	1	.50
Postop NYHA stage	1-4	1 (1.5)	0	1-3	1 (1.2)	0	.43
EuroSCORE II	2-86	14 (20.4)	20	4-40	14.5 (18.6)	23	.75
Vasoactive inotropic score (VIS)	0-450	6	24	0-80	10	16	.44
Echocardiographic findings							
Preop ejection fraction (%)	40-60	60	5	30-60	55	8	**.01**
Preop TAPSE (mm)	13-29	20	4	15-25	19	6	.49
Postop ejection fraction (%)	35-60	57	5	35-60	55	13	.13
Postop TAPSE (mm)	10-21	15	4	9-24	15	6	.75

EuroSCORE II, The European System for Cardiac Operative Risk Evaluation; IQR, interquartile range; NYHA, The New York Heart Association; TAPSE, tricuspid annular plane systolic excursion.

Bold values indicate statistical significance.

**Table 2.** Comparison of Operative Data, Bleeding Amounts, Blood Products, and Intraoperative Medications Between Group A and Group B

	Group A (n = 31) Cell Salvage (+)			Group B (n = 14) Cell Salvage (−)			* P *
	Min-max or n (%)	Median (Mean)	IQR	Min-max or n (%)	Median (Mean)	IQR	
Operative data							
Emergency surgery	13 (41.9)			7 (50)			.61
Coronary artery bypass graft	3 (9.7)			0			.31
Valvular surgery	28 (90.3)			13 (92.9)			.63
Aortic surgery	6 (19.4)			2 (14.3)			.68
Infective endocarditis	4 (12.9)			1 (7.1)			.50
Commando procedure	1 (3.2)			0			.68
Ablation	3 (9.7)			0			.31
Congenital anomaly	2 (6.5)			1 (7.1)			.68
Cross-clamp time (min)	0-308	120 (130)	78	0-170	108 (100)	43	.27
Cardiopulmonary bypass time (min)	78-397	166 (193)	101	115-230	150 (162)	58	.48
Blood products and bleeding amounts							
Intraop red blood cells using	0-5	2 (1.8)	3	0-5	1.5 (1.9)	3	.91
Intraop fresh frozen plasma using	0-3	1 (0.9)	2	0-3	1 (1)	2	.77
Intraop platelet suspensions	0-2	1 (0.6)	1	0-3	1 (0.8)	1	.63
Postop red blood cells using	0-6	0 (0.9)	1	0-3	1 (1)	2	.30
Postop fresh frozen plasma using	0-2	0 (0.2)	1	0-3	0 (0.5)	1	.68
Postop platelet suspensions	0-1	0 (0.06)	0	0-1	0 (0.07)	0	.93
Postop total amount of bleeding (mL)	200-2700	600 (768)	600	150-1900	800 (785)	600	.68
Intraoperative medications							
Dose of tranexamic acid (mg)	0-2000	1000	1000	0-4000	1000	1813	.27
ACT after protamine (sec)	102-185	130	30	77-158	130	19	.50

ACT, activated clotting time; IQR, interquartile range.

**Table 3.** Comparison of Laboratory Parameters Between Group A and Group B

	Group A (n = 31) Cell Salvage (+)		Group B (n = 14) Cell Salvage (−)		* P *
	Min-max or n (%)	Median (IQR)	Min-max or n (%)	Median (IQR)	
Preop laboratory parameters					
White blood cells (10^9^/L)	5-11.7	7.6 (2.1)	1.9-11.5	7.9 (3.2)	.54
Hematocrit (%)	25.6-46.5	39.5 (10.5)	27.5-44.7	36 (8.6)	.41
Platelets (10^9^/L)	140-452	226 (89)	104-418	227.5 (181)	.97
Urea (mg/dL)	17.2-109.2	30.3 (29.4)	14.1-84.3	30.3 (13.8)	.71
Creatinine (mg/dL)	0.54-2.08	0.85 (0.2)	0.51-1.25	0.83 (0.36)	.49
Sodium (mEq/L)	134-147	139 (49	123-145	139.5 (6)	.84
Potassium (mEq/L)	3.6-5.07	4.48 (0.6)	3.41-5.2	4.44 (0.54)	.86
Alanine aminotransferase (IU/L)	6-58	20 (15)	9-301	16.5 (15)	.52
Aspartate aminotransferase (IU/L)	11-97	24 (9)	11-265	22 (12)	.56
C-reactive protein (mg/dL)	0.5-102	4 (13.6)	0.3-38.9	5.4 (10.6)	.59
Troponin T (ng/mL)	3-111	13 (10.4)	6.9-44	13.7 (9.7)	.71
Postop 1st day laboratory parameters					
White blood cells (10^9^/L)	7.5-33.6	20 (12.9)	4.6-26.3	15.8 (5.6)	.06
Hematocrit (%)	24.4-40	30 (5)	24-33.8	29.2 (9.8)	.41
Platelets (10^9^/L)	57-297	151 (98)	92-192	123.5 (64)	.14
Urea (mg/dL)	13.5-110.4	40.7 (23.7)	17.8-62	43.9 (25.4)	.67
Creatinine (mg/dL)	0.54-2.54	1.13-0.62	0.77-2.06	1.09 (0.34)	.63
Sodium (mEq/L)	135-164	143 (7)	133-150	144 (7)	.42
Potassium (mEq/L)	2.9-5.55	4 1 (0.74)	3.69-5.1	4.11 (0.5)	.86
Alanine aminotransferase (IU/L)	10-110	25 (19)	10-302	20 (9)	.07
Aspartate aminotransferase (IU/L)	31-912	83 (48)	33-153	57.5 (41)	**.04**
C-reactive protein (mg/dL)	7.4-189	36 (37.5)	2.9-183	28.6 (29.9)	.41
Troponin T (ng/mL)	138-5945	501 (725)	212-1764	507 (751)	.82

Bold values indicate statistical significance.

**Table 4.** Comparison of Postoperative Data Between Group A and Group B

	Group A (n = 31) Cell Salvage (+)			Group B (n = 14) Cell Salvage (−)			***P***
	Min-max or n (%)	Median (mean)	IQR	Min-max or n (%)	Median (mean)	IQR	
Postoperative exploration	2 (6.5)			0			.47
Cerebrovascular accident	0			0			–
Continuous renal replacement therapy	4 (12.9)			0			.21
Postop atrial fibrillation	5 (16.1)			4 (28.6)			.28
Thoracentesis	2 (6.5)			1 (7.1)			.63
Implantable cardioverter defibrillator	3 (9.7)			1 (7.1)			.68
Gastrointestinal bleeding	0			1 (7.1)			.31
Tracheostomy	1 (3.2)			0			.68
Mortality	6 (19.4)			1 (7.1)			.28
Intubation time (hour)	2-90	13 (17.5)	12	9-72	12 (19.3)	13	.43
Intensive care unit stay (days)	1-30	3 (4.2)	3	2-6	3 (2.8)	1	.46
Hospital stay (days)	1-53	11 (13.7)	12	8-58	13 (16.4)	11	.29

**Table 5.** Comparison of Costs Between Group A and Group B

	Group A (n = 31) Cell Salvage (+)			Group B (n = 14) Cell Salvage (−)			* P *
	Min-max or n (%)	Median (Mean)	IQR	Min-max or n (%)	Median (Mean)	IQR	
Intraop red blood cells cost ($)	0-175	70 (64.3)	105	0-175	52.5 (67.5)	114	.91
Intraop fresh frozen plasma cost ($)	0-63	21 (18.9)	42	0-63	21 (21)	42	.77
Intraop platelet suspensions cost ($)	0-226	113 (76.5)	113	0-339	113 (96.8)	113	.63
Postop red blood cells cost ($)	0-210	0 (33.8)	35	0-105	35 (35)	70	.30
Postop fresh frozen plasma cost ($)	0-42	0 (6.1)	21	0-63	0 (12)	21	.68
Postop platelet suspensions cost ($)	0-113	0 (7.2)	0	0-113	0 (8.07)	0	.93
Intraop blood product costs ($)	0-316	183 (159.8)	233	0-339	175.5 (185.3)	194	.51
Postop blood product costs ($)	0-295	0 (47.2)	56	0-211	35 (55)	75	.27
Cell salvage ($)	241			0			
Total cost ($)	241-831	466 (448)	225	0-520	217.5 (240.4)	221	**<.001**

Bold values indicate statistical significance.

**Table 6.** Cost-Effectiveness of Cell Salvage in Different Studies

**Study**	**Murphy**	**Klein**	**Weltert**	**Xie**	**Wang**	**Ours**
Country	Britain	Britain	Italy	China	China	Türkiye
Year	2005	2007	2012	2014	2018	2025
Cases	30	102	537	72	128	31
Bleeding risk	Low	Low	Low/high	High	Low	High
Cost of RBC($/U)	224	219	154-248	22.8	34	34.8
Cost of autologous blood transfusion ($)	118	153	258	243	184-230	241
Cost-effectiveness	Yes	No	Yes	No	Yes	No
